# Learning Modality Complementary Features with Mixed Attention Mechanism for RGB-T Tracking

**DOI:** 10.3390/s23146609

**Published:** 2023-07-22

**Authors:** Yang Luo, Xiqing Guo, Mingtao Dong, Jin Yu

**Affiliations:** 1 Aerospace Information Research Institute, Chinese Academy of Sciences, Beijing 100094, China; luoyang211@mails.ucas.ac.cn (Y.L.); jinyu@aoe.ac.cn (J.Y.); 2 University of Chinese Academy of Sciences, Beijing 100040, China; 3 Institute of Image Recognition and Machine Intelligence, Northeastern University, Shenyang 110167, China; 2100729@stu.neu.edu.cn

**Keywords:** multi-modality adaptive fusion, mixed-attention mechanism, RGB-T tracking

## Abstract

RGB-T tracking involves the use of images from both visible and thermal modalities. The primary objective is to adaptively leverage the relatively dominant modality in varying conditions to achieve more robust tracking compared to single-modality tracking. An RGB-T tracker based on a mixed-attention mechanism to achieve a complementary fusion of modalities (referred to as MACFT) is proposed in this paper. In the feature extraction stage, we utilize different transformer backbone branches to extract specific and shared information from different modalities. By performing mixed-attention operations in the backbone to enable information interaction and self-enhancement between the template and search images, a robust feature representation is constructed that better understands the high-level semantic features of the target. Then, in the feature fusion stage, a modality shared-specific feature interaction structure was designed based on a mixed-attention mechanism, effectively suppressing low-quality modality noise while enhancing the information from the dominant modality. Evaluation on multiple RGB-T public datasets demonstrates that our proposed tracker outperforms other RGB-T trackers on general evaluation metrics while also being able to adapt to long-term tracking scenarios.

## 1. Introduction

Visual object tracking is an essential branch of computer vision that has recently made significant progress and has been widely applied in various industries, including autonomous driving, intelligent security, and robotics [1]. The current research on visual object tracking predominantly focuses on visible modality (RGB tracking). This is primarily due to the rich color and texture information that RGB images provide, making target feature extraction more feasible as well as enabling the use of relatively low-cost imaging devices based on visible modality [2].

**Figure 1:**
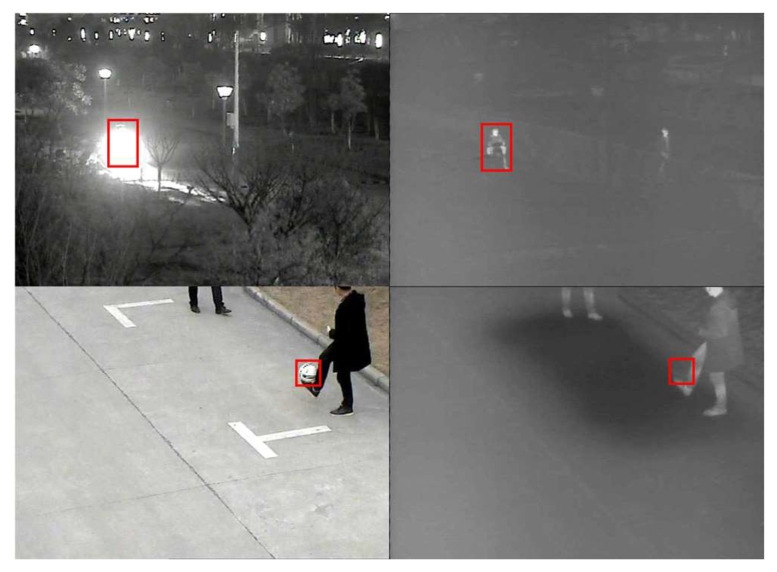
Illustration of the complementary information between multi-modal images.

However, researchers have identified the limitations of RGB tracking in extreme environments such as rain, fog, and low-light conditions. Thermal infrared imaging, on the other hand, relies on the target’s thermal radiation and is less affected by intensity variations in illumination, making it possible to penetrate rain and fog. It provides a complementary and extended modality to RGB tracking [3]. This complementarity has led researchers to progressively concentrate more on object tracking based on the fusion of these two modalities to create trackers with higher accuracy and robustness [4]. As shown in Figure 1, the complementary fusion of visible images and thermal images can provide more stable features for target-tracking tasks to overcome environmental challenges that cannot be dealt with in a single modality.

**Figure 2:**
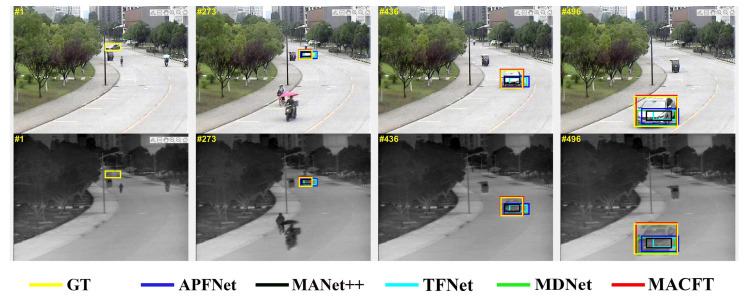
Comparing the model proposed in this paper with other advanced RoI-based RGB-T tracking models when the target undergoes large deformation (the yellow box represents ground truth). We can observe that our MACFT model accurately extracts the features of the target and, due to not being restricted by RoI’s scale, can generate bounding boxes that are closer to the ground truth.

The object tracking method that combines visible and thermal modalities is known as RGB-T tracking. The key point in RGB-T tracking is to achieve complementary information fusion between the visible and thermal modalities. Early RGB-T tracking methods mostly employed CNN for feature modeling. Some methods [5,6] simply concatenated the features extracted from the visible and thermal modalities and fed them into a fully connected network to obtain the target position. However, this simple approach introduced excessive background noise, leading to a decrease in the network’s recognition capability. Other methods [1,7,8,9,10,11,12,13,14,15] first generated candidate boxes (RoIs) from the search frame and then performed feature fusion on the RoIs from different modalities using mechanisms such as gating, attention, and specific data attribute annotations. Finally, binary classification of foreground/background was conducted on the fused features, and the bounding box was regressed. One significant drawback of such methods is that the sizes and ratios of RoI regions are limited and local in nature. They cannot adapt flexibly to variations in the target’s appearance, as illustrated in Figure 2. Additionally, these methods fail to incorporate sufficient background information for feature learning. This limitation results in inadequate modeling of the global environmental context in the search framework due to the potential insufficient feature interaction between RoIs from different modalities. Consequently, the mutual enhancement and complementary effects between the two modalities are restricted.

To enhance the adaptive fusion capability of the algorithm for complementary information from different modalities, we have designed a tracking architecture based on a combination of multiple attention mechanisms (referred to as MACFT). This architecture can learn the complementary features between visible and thermal modalities. The core of the architecture is the modality shared-specific feature interaction (SSFI) module. The SSFI module performs cross-attention and mixed-attention operations on the shared and specific features from both the visible and thermal modalities. The cross-attention operation facilitates the interaction of information between modalities, while also merging the shared and specific features to suppress the weights of low-quality modalities. The mixed-attention operation is used to enhance the fused modality information and further remove redundant noise.

Furthermore, in the feature extraction stage, considering that the effective receptive field of CNN may grow much more slowly in practice than theoretically due to the continuous downsampling process [16], there is still a significant amount of local information retained even after multiple convolutional layers. To achieve long-range modeling of target pixels, we employ the Vision Transformer [17] architecture, which has been widely used in recent years for Single-Object Tracking (SOT), as the backbone of our network. We extend it to a dual-branch Siamese architecture. Additionally, although visible and thermal images have different imaging wavelengths, they still share many correlations, such as object edges, contour information, and fine-grained texture details [9]. Therefore, apart from the dual-branch backbone used to extract features from visible and thermal images separately, we introduce a shared-parameter backbone. By introducing a KL divergence loss function as a constraint, we maintain the consistency between modalities and learn shared features across modalities. Finally, we insert the SSFI module after the backbone to perform feature fusion and achieve cross-modal interaction.

After cross-fusing features from the modality-specific feature branch and the modality-shared feature branch, we generate the target bounding box with only a simple structured corner point predictor. After extensive experiments on several public RGB-T tracking datasets, we verify that the proposed method is effective and outperforms most of the current state-of-the-art RGB-T tracking models.

The main contributions of this paper are summarized as follows. Firstly, a high-performance RGB-T tracking method has been proposed, which utilizes a deep transformer-based backbone to extract both the specific features and shared features from visible and thermal modality images. This method demonstrates excellent performance in challenging scenarios such as significant target deformations and occlusions. Secondly, a novel modality feature fusion structure based on a combination of multiple attention mechanisms is designed, and through the cross-input and fusion of cross-modal shared/specific information, the complementary features between modalities can be fully interacted. It can adaptively suppress low-quality modalities and enhance the features of dominant modalities for tracking. Thirdly, as a versatile fusion strategy, the module we designed can be easily extended to other multi-modal scenarios.

## 2. Related Work

### 2.1. RGB Tracking Methods

Visual object tracking based on RGB images has been developed for quite some time, but it was not until recent years when deep neural networks were successfully applied to computer vision tasks that models with high tracking accuracy emerged. They can be broadly classified into two categories, namely, discriminative models and generative models.

Discriminative models are trained using the whole image labeled with the target location, aiming to obtain a classifier that can distinguish between foreground and background. They often need to generate multiple proposal regions during the tracking process before feeding the classifier through the forward propagation to obtain positive and negative samples. These samples are further refined through post-processing methods such as the cosine window penalty, and the best samples are retained. Typical discriminative trackers include MDNet [8], ATOM [18], DiMP [6], etc. Although these models are known for their high accuracy, the excessive online processing required for them consumes many computational resources, which results in slow operation; indeed, the RGB-T tracking methods extended from the above-mentioned approaches also face the challenge of not being able to meet real-time requirements, such as DMCNet [12], MaCNet [3], APFNet [1], etc. To address this drawback, Jung et al. [19] modified the forward propagation based on MDNet with the ROI Align [20], thereby increasing its speed.

Contrary to the discriminative model, the generative model solely relies on offline training and has no online update step. The final prediction of the target is obtained by computing the joint probability density of both the template and search image. Typical generative models such as the Siamese network-based tracker [21,22,23]. The generative model’s major strength is its small computational effort and fast speed, but its tracking accuracy is often inferior to that of discriminative models due to insufficient information interaction between the template and the search images and the lack of an online update step.

In recent years, the field of computer vision has witnessed the gradual replacement of CNN as the backbone architecture with the transformer. In the domain of visual object tracking, Chen et al. [24] designed self-contextual augmentation (ECA) and cross-feature augmentation (CFA) modules in their proposed model TransT that differ from the traditional transformer structure to achieve a better fusion of features between templates and search regions. Yan et al. [25] proposed a method based on the transformer codec structure to achieve tracking directly by bounding box prediction, in which the image is fed into a simple fully convolutional network for feature extraction and then directly fed into the transformer prediction head for bounding box prediction without pre-generating proposal regions. Cui et al. [26] proposed an asymmetric attention mechanism for information interaction between the template and search images, which saves computational resources and provides the possibility to update the template online during the tracking process. Furthermore, attention mechanisms have also been widely utilized in other fields. For instance, Zhou et al. [27] and Zheng et al. [28] applied attention mechanisms in tool condition monitoring and bearing defect detection.

To improve the speed of our tracker, the model proposed in this paper is based on the idea of the generative model, whereby the process of tracking relies entirely on offline training. Inspired by the work of Chen et al. [29], the full interaction between the target template and the search image is implemented within the transformer backbone, which also gives our tracker a very high accuracy.

### 2.2. RGB-T Tracking Methods

RGB-T tracking, based on the fusion of visible and thermal modalities, is a branch of single-object tracking that focuses on effectively utilizing complementary information from multiple modalities to achieve a more stable and robust tracking performance. In 2018, Wang et al. [30] introduced correlation filters into the field of RGB-T tracking, using average correlation peak energy to compute weights. In 2021, Zhang et al. [31] proposed a novel robust RGB-T tracking framework called JMMAC. JMMAC is based on the well-known ECO algorithm [32] in single-modality tracking and takes motion principles into full consideration to design an adaptive structure that allows the model to switch between appearance modeling and motion modeling.

In recent years, more high-performance RGB-T trackers have been proposed because of the introduction of deep neural networks. Li et al. [10] developed a multi-adapter convolutional network for RGB-T tracking by following MDNet’s multi-domain learning framework that performs feature extraction for modal-generic features, modal-specific features, and instance features. However, performing forward propagation too many times leads to slow running speed of the model, which cannot meet the real-time requirements. Zhu et al. [14] also used MDNet, but through a 1 × 1 convolutional kernel + Relu + LRN (local response normalization) to form a feature aggregation module to fuse the features of two modalities and randomly reject the feature channels using the channel dropout technique, which effectively eliminates redundant information and noise while preventing model overfitting. Zhang et al. [5] first implemented the task of RGB-T fusion tracking based on a Siamese network combined with multi-modal features. In this method, the modality weight calculation is designed according to the size of the predicted target position deviation, which improves the reliability of the model. The disadvantage of this method is that only the concatenation method is used for the fusion of modes, which fails to effectively suppress low-quality mode noise. Zhu et al. [7] proposed a trident architecture to integrate the fused modality features and two modality-specific features, thus achieving robust target representation. Zhang et al. [33] introduced a complementary perception module for multi-modal feature fusion, which reduces the modality discrepancy between single-modal features to enhance the discriminability of fused features. This is done to fully utilize training data and address various challenges such as illumination variations, occlusion, thermal crossover, and fast motion. Zhu et al. [11] designed a feature fusion structure based on the attention mechanism proposed in SKNet [34] during the modality fusion stage, enabling different neurons of the model to have adaptive receptive fields. Zhang et al. [35] built upon the mfDiMP [36] framework and unified multiple modality fusion strategies (including pixel-level fusion, feature-level fusion, and decision-level fusion) into a hierarchical fusion framework. They also designed a self-attention-based module to model the confidence of modalities and exploit modal information in a non-local manner.

With the trend of the transformer architecture surpassing CNN in performance in recent years, many researchers in the field of RGB-T object tracking have attempted to incorporate the core idea of the self-attention mechanism into their algorithm models. One typical example is APFNet proposed by Xiao et al. [1]. Building upon the challenges posed by RGB-T-specific attributes, APFNet adopts specific strategies for fusion and incorporates three independent and separate encoders (based on the self-attention mechanism) and decoders (based on the cross-attention mechanism) in each fusion branch. This enables self-enhancement within modalities and interaction enhancement between modalities. The limitation of this method is that it still relies on CNN to extract general features and performs attention operations in the high-level semantic space after downsampling multiple times but fails to consider more fine image information. Zhu et al. [37] introduced ViPT, which is based on a unified ViT backbone for feature extraction. By leveraging the idea of visual prompt tuning [38], ViPT adds the thermal modality information to the tokens for learning, while preserving the original feature structure of the transformer. This approach significantly reduces the number of trainable parameters, but the price is insufficient fusion, making the model perform poorly in scenes such as illumination changes.

## 3. Method

**Figure 3:**
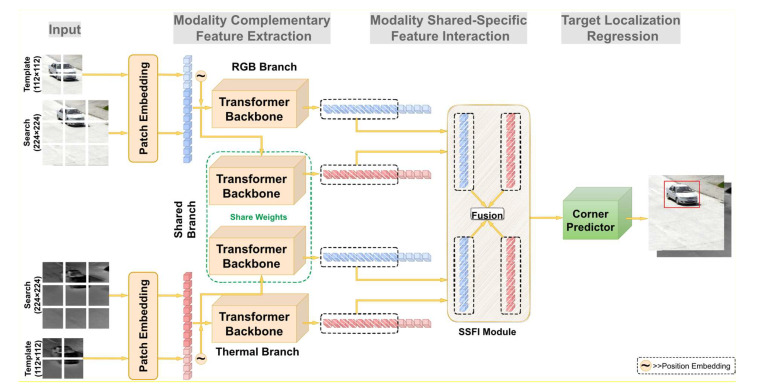
An overview of the MACFT model, which is divided into three parts, which are used for modal-specific/shared feature extraction, information fusion between modalities, and bounding box regression.

In this section, our proposed RGB-T tracking model MACFT is described in detail, as shown in Figure 3. The whole model consists of three parts, which comprise the modality complementary feature extraction network based on the transformer backbone, the modality shared-specific feature interaction module based on the mixed-attention mechanism, and the target localization regression network; each of these components will be discussed more specifically later. The overall information pipeline of the model can be summarized as follows: after the images from the two modalities are sent to different feature extraction backbones, the modal-shared features and modal-specific features will be obtained, respectively, and then these features will be crossed in pairs and fused by the SSFI module (generating a group of shared features of visible mode and specific features of thermal mode, and a group of shared features of thermal mode and specific features of visible mode). The fused features are then fed into the target localization network to generate the position of the target. Additionally, the explanation of symbols used in this section will be shown in Abbreviations section.

### 3.1. Transformer-Based Complementary Feature Extraction Backbone

In our proposed model, the feature extraction part is divided into four branches, namely, the visible modal-specific feature extraction branch, the thermal modal-specific feature extraction branch, and two modal-shared feature extraction branches. ViT [17] served as the base backbone for all four branches, initiated with parameters from the CLIP [39] pre-trained model. Furthermore, the template image is concatenated with the search image in the same dimension to achieve information interaction. The process is detailed as follows.

#### 3.1.1. Model-Specific Feature Extraction Branch

First, given a video sequence, we select the first frame as the reference frame and crop the template image $Z\in R^{H_{z}\times W_{z}\times 3}$ according to the labeled bounding box, where $H_{z}\times W_{z}$ is the size of the template image, and accordingly, the subsequent frames in the video sequence are used as the search image with the size set to $X\in R^{H_{x}\times W_{x}\times 3}$. To accommodate the generic ViT backbone, we set the search image size ${\;H}_{x}\times W_{x}$ to 224 × 224 and the template image size $H_{z}\times W_{z}$ to 112 × 112.

Before using the transformer backbone for feature extraction, the input image is first serialized, i.e., patch embedded, by dividing the 2D image into N blocks of size ${D=P}^{2}\times C$ (*P* is the size of patch; here, *P* = 16 since the backbone used is ViT-B-16, and *C* is the number of channels of the image (here *C* = 3), before projecting the patch onto the space with dimension *D* through a linear transformation. For our input template image and search image, the dimensions of the image after serialization will be transformed into $Z_{p}\in R^{N_{z}\times D}$ and $X_{p}\in R^{N_{x}\times D}$, where $N_{z}=H_{z}W_{z}/P^{2}$ and $N_{x}=H_{x}W_{x}/P^{2}$.

For the transformer backbone, there is an additional step in the input process which is to add positional encoding to help the model distinguish the order of the input sequence. For the search image sequence, the pre-trained positional encoding $p_{s}=p_{ptr}$ can be used directly because its size is consistent with the pre-trained ViT backbone input. However, for the template image, its size is not aligned with the ViT input and cannot accommodate the pre-trained positional encoding, so we set up a learnable positional encoding structure that consists of a two-layer fully connected network, which is added to the template image sequence to obtain the positional encoding vector $p_{z}$ of the template image.

After adding the position encoding vector, we obtain the final input vectors $z\in R^{N_{z}\times D^{*}}$ and $x\in R^{N_{x}\times D^{*}}$, and subsequently, we concatenate z,x along the first dimension and feed them into the transformer backbone network for learning. Let the concatenated vector be r:

(1) $$r=Concat\left(z,x,dim=0\right).$$

Let the output of layer n in the backbone network be $r^{n}$; then we have:

(2) $$\begin{array}{c} \left[r^{*}\right]=\left[r^{n-1}\right]+MA\left(\left[r^{n-1}\right]\right),\\ \left[r^{n}\right]=\left[r^{*}\right]+FFN\left(\left[r^{*}\right]\right). \end{array}$$

where MA denotes mixed-attention operation and FFN denotes feed forward network. The calculation within MA can be expressed as follows:

(3) $$\mathrm{MA}\left(\left[r^{n}\right]\right)=softmax\left(\left[\begin{array}{c} a(z^{n},z^{n}),a(z^{n},x^{n})\\ a(x^{n},z^{n}),a(x^{n},x^{n}) \end{array}\right]\right)\left(\left[\begin{array}{c} z^{n}W_{V}\\ x^{n}W_{V} \end{array}\right]\right)$$

where $a(x,y)=(xW_{Q}){\left(yW_{K}\right)}^{T}/\sqrt{d}$.

It is in the cross-attention operations such as $a\left(z^{n},x^{n}\right)$ and $a(x^{n},z^{n})$ that the model learns the relationship between the template image and the search image, and in the self-attention operations such as $a(z^{n},z^{n})$ and $a(x^{n},x^{n})$ that this relationship is self-enhanced. We construct two identical and independent feature extraction branches for RGB and thermal images based on the above method for extracting features unique between different modalities. Let the feature vectors output from the two branches be:

(4) $$R_{v}\in R^{{(N}_{z}+N_{x})\times D^{*}},\;R_{t}\in R^{{(N}_{z}+N_{x})\times D^{*}}.$$

#### 3.1.2. Modal-Shared Feature Extraction Branch

To better construct a complementary feature representation between the visible and thermal modalities, we add two modal-shared feature extraction backbones to the model (again using ViT as the backbone with the same input structure as the modal-specific feature branch) for learning features shared by both modalities. Further, to be able to constrain the backbone of this branch to ensure that its output features are consistent across the two modalities, we introduce a Kullback–Leibler (KL) divergence loss $L_{div}$. The formula is expressed as follows:

(5) $$L_{div}=KL(G_{v}\|G_{t})=\frac{1}{N_{z}+N_{x}}\sum_{n=1}^{N_{z}+N_{x}}\left(g_{vn}log(g_{vn}-g_{tn})\right)$$

where $G_{v}\in R^{{(N}_{z}+N_{x})\times D^{*}}$, $G_{t}\in R^{{(N}_{z}+N_{x})\times D^{*}}$ denote the output vectors (after *softmax*) of the visible modality input and thermal modality input through the shared feature branch backbone, respectively, while $g_{vn},\;g_{tn}$ denote the nth term of the first dimension in $G_{v},G_{t}$. KL divergence is based on the information entropy principle, and it can accurately measure the information lost when approximating one distribution with another, so it can be used to calculate the similarity between two distributions.

### 3.2. Modality Shared-Specific Feature Interaction Module

After obtaining the features from the four branches (the visible branch, the thermal branch, and the shared branches), we design a modality shared-specific feature interaction module based on the mixed-attention mechanism, with the aim of learning an adaptive discriminative weight for features from different modalities, thus enhancing high-quality modality while suppressing low-quality modality.

**Figure 4:**
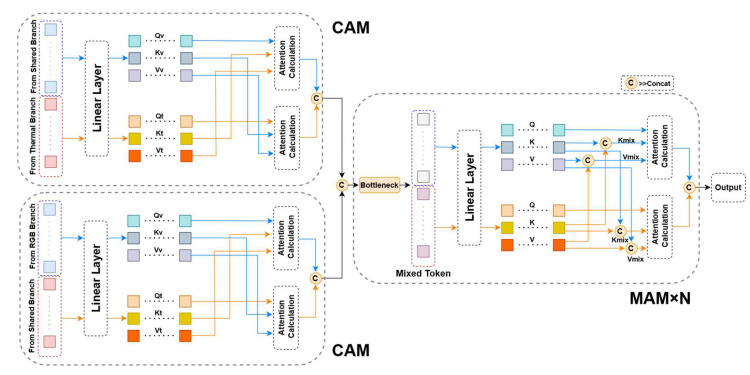
Schematic diagram of the SSFI module. It consists of 2 CAM modules and N = 6 MAM modules. The CAM modules utilize cross-attention to aggregate features from the visible specific/thermal shared branch and the thermal specific/visible shared branch. The MAM modules further enhance the aggregated features by introducing mixed-attention.

As shown in Figure 4, we use two types of attention mechanisms in the SSFI module: the cross-attention module (CAM) and the mixed-attention module (MAM).

Given a vector consisting of visible features connected with thermal features $x=Concat(x_{v},x_{t})$, we first map the input into three equally shaped weight matrices called *query*, *key*, and *value* by a linear layer. $q_{v},\;{\;k}_{v},\;v_{v}$ are used below to denote the visible part and $q_{t},\;{\;k}_{t},\;v_{t}$ denote the thermal part. From (3), the calculation of MAM can be described as:

(6) $$\mathrm{MAM}\left(\left[x\right]\right)=softmax\left(\left[\begin{array}{c} a(x_{v},x_{v}),a(x_{v},x_{t})\\ a(x_{t},x_{v}),a(x_{t},x_{t}) \end{array}\right]\right)\left(\left[\begin{array}{c} x_{v}v_{v}\\ x_{t}v_{t} \end{array}\right]\right)$$

where $\;a(x_{v},x_{t})=(x_{v}q_{v}){\left(x_{t}k_{t}\right)}^{T}/\sqrt{d}$,$a(x_{v},x_{v})$, $a(x_{t},x_{t})$, $a(x_{t},x_{v})$ in the same way.

The CAM module, on the other hand, eliminates the self-attention component and retains only the cross-attention, which can be described as:

(7) $$\mathrm{CAM}\left(\left[x\right]\right)=\left[\begin{array}{c} \mathrm{softmax}\left(\left.\begin{array}{c} a\left(x_{v},x_{t}\right) \end{array}\right.\right)*\left.\left.\begin{array}{c} x_{t}v_{t} \end{array}\right.\right.\\ \mathrm{softmax}\left(\left.\begin{array}{c} a\left(x_{t},x_{v}\right) \end{array}\right.\right)*\left.\left.x_{v}v_{v}\right.\right. \end{array}\right]$$

In the SSFI module, we connect the outputs from the visible modal-specific feature branch with the modal-shared feature branch (thermal part), and from the thermal modal-specific feature branch with the modal-shared feature branch (visible part), but only the part with the same size as the search image is selected and the template part is discarded:

(8) $$\begin{array}{c} R_{v},R_{t}\in R^{{(N}_{z}+N_{x})\times C}\to R_{v}^{x},R_{t}^{x}\in R^{N_{x}\times C},\\ \;G_{v},G_{t}\in R^{{(N}_{z}+N_{x})\times C}\to G_{v}^{x},G_{t}^{x}\in R^{N_{x}\times C}. \end{array}$$

(9) $$\begin{array}{c} H_{vt}=Concat\left(R_{v}^{x},G_{t}^{x}\right),\\ H_{tv}=Concat\left(R_{t}^{x},G_{v}^{x}\right). \end{array}$$

Subsequently, $H_{vt},H_{tv}$ are fed into the CAM module for modal information interaction:

(10) $$\begin{array}{c} {∁}_{tv}=CAM\left(H_{tv}\right),\\ {∁}_{vt}=CAM\left(H_{vt}\right). \end{array}$$

Only cross-attention is used here for the fusion of features from different branches due to the fact that sufficient self-enhancement has been performed in the backbone for the features from each modality, and there is no need to add extra self-attention modules. Additionally, removing the self-attention operation can also reduce the model’s parameter count. However, in the following MAM modules, we introduce self-attention operation because the initial interaction mapped features from different modalities into a new space, necessitating further enhancement of feature representation with the self-attention mechanism. These points will be supported in Chapter 4 in the subsection on ablation studies.

Finally, we connect the output of the CAM module and reduce the dimension and send it to the MAM module to further enhance the information interaction between the modalities:

(11) $${∁}_{mix}=\;DR\;(Concat\;({∁}_{tv},{∁}_{vt})).$$

(12) $${Ϻ}_{mix}=\;\mathrm{MAM}\;({∁}_{tv},{∁}_{vt}).$$

(13) $$Output=\;DR\;({Ϻ}_{mix}).$$

### 3.3. Target Localization Regression Network

After the SSFI module, we use a lightweight corner predictor to localize the target. First, the features are reshaped into 2D; then, for the top left and bottom right corners of the bounding box, the features are downsampled using 5 convolutional layers, and finally the corner coordinates are calculated using the *softArgmax* layer and are reverse mapped to the coordinates corresponding to the original image according to the scale of the image crop.

### 3.4. Training Method

#### 3.4.1. Training in Stages

We employ a staged approach for model training, which comprises three stages. The first two stages involve backbone training, requiring the modality fusion network to be removed while retaining only the corner predictor.

In the first stage, we freeze the modal-shared feature branch and load the pre-trained model to initialize the backbone of the modal-specific feature branches. We then train the backbone of the corresponding branches using RGB and thermal image data and save the model parameters.

In the second stage, the modal-specific feature branches are frozen, and the modal-shared branch is unfrozen. During the forward pass, RGB and thermal images are repeatedly used to update the parameters of the backbone, and the model parameters are saved.

In particular, during the training of the feature extraction backbone in the first two stages, in order to reduce the number of trainable parameters of the network and considering the shared characteristics of the low-level features between images, we freeze the first eight multi-head attention modules of the ViT backbone and only fine-tune the last four.

In the third stage, all feature extraction backbone branches are frozen, and the models trained in the first two stages are loaded for initializing the backbone parameters. The modality fusion network (SSFI) is added for training and the model parameters are saved.

#### 3.4.2. Loss Function

The model proposed in this paper uses a total of three loss functions in the offline training phase, which are *Ɩ*_1_ loss, generalized GioU loss [40], and KL divergence loss, and the loss functions are combined in the first and third stages of model training in the following manner:

(14) $$L=\lambda_{\mathrm{giou}\;}L_{giou}\left(b_{i},b_{i}^{*}\right)+\lambda_{L_{1}}L_{1}\left(b_{i},b_{i}^{*}\right).$$

where $L_{1}$ denotes the *Ɩ*_1_ loss, $L_{giou}$ denotes the generalized GIoU loss, $b_{i}$ denotes the target bounding boxes predicted by the model on the search image, $b_{i}^{*}$ denotes the true value of the target bounding boxes, and $\lambda_{\mathrm{giou}\;}$ and $\lambda_{L_{1}}$ are the weight parameters of the loss.

In the second stage of model training, we additionally introduce the KL divergence loss, and the loss function is combined in the following way:

(15) $$L=min[\lambda_{\mathrm{giou}\;}L_{giou}\left(b_{v},b_{v}^{*}\right)+\lambda_{L_{1}}L_{1}\left(b_{v},b_{v}^{*}\right),\;\lambda_{\mathrm{giou}\;}L_{giou}\left(b_{t},b_{t}^{*}\right)+\lambda_{L_{1}}L_{1}\left(b_{t},b_{t}^{*}\right)]\;+\lambda_{KL}L_{div}\left(b_{v},b_{t}\right).$$

where $L_{div}$ denotes KL divergence loss, $\lambda_{KL}$ is its weight, and $b_{v}$*,* $b_{t}$ represent the target bounding boxes obtained from the RGB image and thermal image after modal-shared feature branches and corner predictors, respectively.

### 3.5. Inference

The RGB-T tracking model proposed in this paper does not contain any additional post-processing operations in the inference stage, and consists of only a few steps of image sampling, forward pass, and coordinate transformation, so MACFT can run in real time at a high speed.

## 4. Experiments

This section focuses on the experimental setup and procedure, evaluated on three major RGB-T public datasets (RGBT234 [41], LasHeR [42], and VTUAV [35]) and compared with current state-of-the-art models.

### 4.1. Implementation Details

#### 4.1.1. Experimental Platform Setup

MACFT training was performed in Python 3.8.13, Pytorch 1.7.1, and CUDA 11.7. All experiments were conducted on a workstation equipped with two RTX 3090 GPUs, a Core i7 12700k CPU, and 32 GB of RAM.

#### 4.1.2. Parameter Details

The first stage of training trains visible and thermal modal-specific feature branches for 40 epochs, which was in total an 80-epoch training: 40 epochs of training for the second stage and 80 epochs of training for the third stage. Each epoch consists of 12,000 image samples, with a batch size of 64. All models are learned using the AdamW optimizer [43], with the weight decay set to 10^−4^ and the initial learning rate set to 10^−5^ for the backbone and 10^−4^ for the rest.

In particular, when testing the LasHeR dataset, the model is trained using LasHeR’s own training set, while when testing the RGBT234 dataset and VTUAV dataset, the model is trained using the full LasHeR dataset. In all training stages, similar to our baseline model, $\lambda_{\mathrm{giou}\;}$ and $\lambda_{L_{1}}$ are set to 2 and 5, respectively, whereas in the second stage of training, $\lambda_{KL}$ is set to a large number (in this case, 800) with the purpose of suppressing the optimizer’s focus on other loss terms, thereby facilitating the learning of modal-shared features.

### 4.2. Public Dataset Evaluation

#### 4.2.1. Evaluation Metrics

In this paper, we use the two most commonly used metrics to evaluate the performance of the tracking algorithm, which are the precision rate (PR) and the success rate (SR). The precision rate is calculated as the center location error (CLE) between the predicted bounding box and the ground truth, which is eventually expressed as the percentage of frames where the CLE is within a threshold (usually set to 20 pixels), and by varying the size of the threshold, a precision rate curve can be obtained. The success rate is calculated as the percentage of frames whose intersection over union (IoU) between the predicted bounding box and the ground truth is greater than the threshold value, and by setting different thresholds, a success rate curve can be obtained.

#### 4.2.2. Evaluation on RGBT234 Dataset

The RGBT234 dataset contains a total of 234 RGB-T video sequences with a total of about 116.7 K frames, as well as 12 different challenge attributes, no division between the training and test sets, and the images of the two modalities are not precisely aligned. As shown in Table 1, we evaluate 10 different RGB-T tracking algorithms (APFNet [1], TFNet [7], DAFNet [13], MANet++ [9], MANet [10], SiamCDA [33], ViPT [37], JMMAC [31], MaCNet [3], DMCNet [12]) on the RGBT234 dataset and compare them with the algorithm proposed in this paper. The results show that our MACFT model achieves a precision rate of 85.7% and a success rate of 62.2% on the RGBT234 dataset, which is 5.7% and 4% better than the baseline, respectively.

#### 4.2.3. Evaluation on LasHeR Dataset

**Figure 5:**
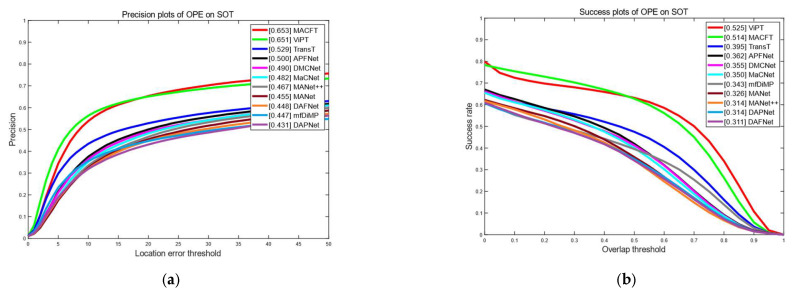
Evaluation results on the LasHeR dataset; (**a**) is the precision rate curve, (**b**) is the success rate curve.

The LasHeR dataset is the largest RGB-T tracking dataset with accurate annotation and alignment, with a total of about 734.8 K frames, 19 challenge attributes, divided training and testing sets, and a higher tracking difficulty compared to the RGBT234 dataset. To further evaluate the effectiveness of the model proposed in this paper, as shown in Figure 5, we compare its results evaluated on the LasHeR dataset with 10 RGB-T tracking algorithms (APFNet [1], DMCNet [12], MaCNet [3], mfDiMP [36], MANet [10], MANet++ [9], DAPNet [14], DAFNet [13], TransT [24], ViPT [37]). Our MACFT model achieves a 65.3% precision rate and a 51.4% success rate on the LasHeR dataset, which is a 7.5% and 5% improvement, respectively, compared to the baseline.

#### 4.2.4. Evaluation on VTUAV Dataset

The VTUAV dataset is a large-scale RGB-T dataset designed for unmanned aerial vehicle (UAV) tracking, and it is currently the highest-resolution RGB-T dataset that considers both short-term and long-term tracking scenarios. However, the disadvantage of VTUAV is that the visible and thermal modalities are not aligned, and each frame in the sequence is not precisely manually annotated. Nevertheless, from another perspective, such data are more in line with the images obtained in real-world applications.

We evaluated our approach on both short-term and long-term tracking subsets of the VTUAV dataset and compared it with state-of-the-art RGB-T tracking algorithms. The results are shown in Table 2 and Table 3. In particular, HMFT-LT is a model optimized specifically for long-term tracking (using GlobalTrack [44] as the global detector and RT-MDNet [19] as the tracking selector). When RT-MDNet recognizes that the target does not exist, GlobalTrack is selected to search for the target in the entire image.

In the short-term subset evaluation, MACFT achieves a precision rate of 80.1% and a success rate of 66.8%, far surpassing the second-ranked tracker. In the long-term subset evaluation, MACFT achieves a precision rate of 54.1% and a success rate of 46.7%. It can be observed that even without specific optimization for long-term tracking tasks, MACFT still achieves state-of-the-art tracking performance on the long-term subset, which also demonstrates its potential for long-term tracking tasks.

#### 4.2.5. Evaluation Based on Challenge Attributes

To better evaluate the effectiveness of our proposed MACFT model, we evaluated it on the RGBT234 dataset based on 12 labeled challenge attributes, which are: No Occlusion (NO), Partial Occlusion (PO), Heavy Occlusion (HO), Low Illumination (LI), Low Resolution (LR), Thermal Crossover (TC), Object Deformation (DEF), Fast Motion (FM), Scale Variation (SV), Motion Blur (MB), Camera Movement (CM), and Background Clutter (BC).

As shown in Table 4, our proposed model (MACFT) outperforms other state-of-the-art tracking models (DMCNet [12], APFNet [1], TFNet [7], MaCNet [3], DAFNet [13], ViPT [37], JMMAC [31], respectively) in most challenging attributes, especially in scenarios involving object occlusion and deformation, which further validates the effectiveness of the tracking model.

#### 4.2.6. Qualitative Evaluation

**Figure 6:**
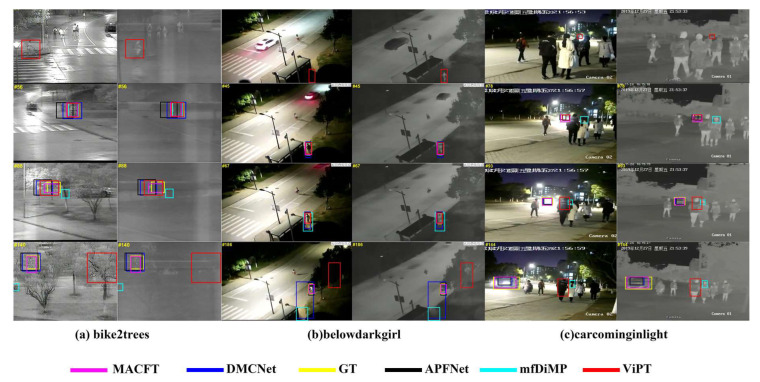
A visual example of the effectiveness of our proposed tracking method. (**a**–**c**) are three challenging sequences selected from the LasHeR dataset, and also show the tracking results of other advanced RGB-T trackers for comparison.

Our results for the tracking performance of MACFT on several challenging video sequences are presented in Figure 6. These sequences included object deformation, camera movement, object occlusion, low illumination, high illumination, and other challenges. To test our method, we compared it with several advanced RGB-T trackers. This comparison indicates that MACFT had a higher ability to utilize the modality complementary information while excluding the interference of low-quality modality. Furthermore, MACFT had a more accurate understanding of the high-level semantic features of the target, making it more robust in tracking.

#### 4.2.7. Comprehensive Evaluation of Efficiency and Effectiveness

**Figure 7:**
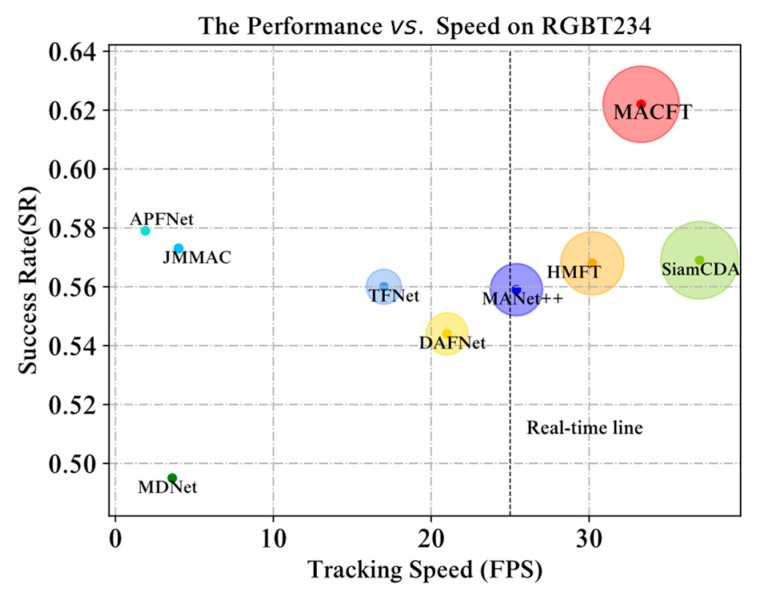
Comprehensive comparison of tracking success rate and speed on the RGBT234 dataset. Our MACFT model achieves the highest tracking success rate under the premise of ensuring real-time running (33.3 FPS on average).

Our method runs at 33.3 FPS (50.2 FPS for a lighter version with modality-shared feature branches removed) on an RTX 3090 GPU and can run in real time. Figure 7 gives a visual display of the comprehensive comparison of MACFT’s success rate and running speed on the RGBT234 dataset with other advanced RGB-T tracking methods (the compared methods include TFNet [7], APFNet [1], MANet++ [9], MDNet [8], JMMAC [31], HMFT [35], SiamCDA [33], and DAFNet [13]).

### 4.3. Visualization

**Figure 8:**
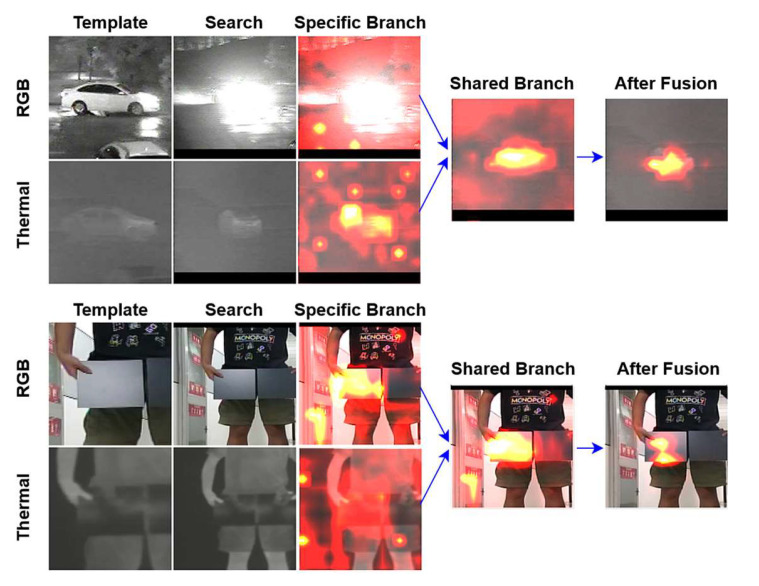
Visualization of the attention weights of different branches when one of the modalities is of low quality.

To prove that the fusion structure we designed can suppress the noise well from low-quality modality while enhancing the feature expression of the dominant modality, we selected specific sequences for visualization. The results are shown in Figure 8.

The visualization method involves computing the attention weights by multiplying the query weight matrix with the key weight matrix at different network positions. Then, the average is taken along the token dimension to obtain a 14 × 14 (search size/patch size) attention weights map, which represents the regions of the image that the network focuses on at the current position.

We selected the car light sequence (thermal modality is dominant) from the RGBT234 dataset and the left mirror sequence (visible modality is dominant) from the LasHeR dataset for visualization. Among the attention weights maps, we can clearly observe that when facing a lack of target information in a certain modality, the network struggles to concentrate its attention entirely on the target location after feature extraction by the backbone. However, after incorporating our designed fusion structure, the noise introduced from the low-quality modality image is effectively suppressed, while the features from the dominant modality image are further enhanced.

### 4.4. Ablation Study

#### 4.4.1. Model Pruning Experiments

To verify the validity of our model structure, specific analyses were performed for each component, and we set up several variants of the MACFT model (prefix B for baseline and DM for dual-modality), namely (1) MACFT (B-T): uses the baseline model + thermal modality, (2) MACFT (B-RGB): uses the baseline model + visible modality, (3) MACFT (DM): uses 2 modal-specific branches but removes all fusion modules based on attention mechanism, only concatenating the features from the two modalities and sending them to a fully connected layer for fusion, (4) MACFT (DM + CAM): uses 2 modal-specific branches (removing modal-shared feature branch) and 6 cascaded CAM modules for modality fusion (5) MACFT (DM + MAM): uses 2 modal-specific branches (removing modal-shared feature branch) and 6 cascaded MAM modules for modality fusion, (6) MACFT (DM + CAM + COM): uses all three branches and uses CAM modules in early feature fusion, but in late feature fusion, uses CAM modules instead of all MAM modules, (7) MACFT (w/o-FT): uses the complete model of MACFT but removes the finetune operation of the backbone.

The performance of the above control groups on the two RGB-T datasets is shown in detail in Table 5. The experimental results show that each component in MACFT improves the tracking performance to different degrees, and also indicates that the designed modal-shared branch and modality fusion network are effective.

In particular, comparison between MACFT (DM + MAM) and MACFT (DM + CAM) confirms our view that since the features from different modalities are sufficiently self-enhanced in the backbone, it does not make sense to simply add a self-attention operation after the backbone without introducing the modal-shared branch. However, after introducing modal-shared feature branches and cross-fusing them with modal-specific branches, the feature distribution is changed so there is room for learning with the introduction of the self-attention operation.

#### 4.4.2. Parameter Tuning Experiments

To find the optimal number of MAM modules in series under the current volume of datasets, we evaluated the precision rate and success rate on the LasHeR test set and conducted experiments on the number of MAM modules in series from 4 to 8, respectively, and the results are shown in Table 6. The best results are obtained with 6 MAM modules in series under the current volume of training datasets and using fewer MAM modules is not enough to build the relationship of complementary information interactions between modalities, while using more MAM modules makes the model parameters not adjusted properly under the same epoch of training due to the limitation of the volume of data.

#### 4.4.3. Parameter Size and Inference Speed

As an object tracking algorithm, the inference speed of MACFT is crucial as it directly affects the practical usability of the algorithm. Table 7 shows the inference speed of MACFT and its variants on an RTX 3090 GPU, as well as the success rate of tracking on the LasHeR dataset, and the number of trainable parameters for these models.

It can be observed that the inclusion of the modal-shared branch has a significant impact on the inference speed. If the requirement for tracking performance is not very high but the demand for inference speed is high in practical applications, a light version without the modal-shared branch can be considered.

## 5. Conclusions

In this paper, we propose an RGB-T tracking method, MACFT, based on a mixed-attention mechanism. Four separate branches are established to extract modal-specific features and modal-shared features, and information interaction between the template image and the search image is introduced in each layer of the backbone. In the modality fusion stage, both cross-attention and mixed-attention are used to fuse the image features from different branches, which effectively reduces the noise from low-quality modality and enhances the feature information of the dominant modality. After conducting extensive experiments, we demonstrate that MACFT can understand the high-level semantic features of the target well, thus enabling it to cope with multiple challenges and achieve robust tracking.

However, our work still has some limitations, such as insufficient discriminability of target instances, large parameter count, and complex training procedures. In the future, we will address these issues by exploring various approaches, including but not limited to designing lightweight fusion modules, employing knowledge distillation to reduce parameter count, and integrating modal feature fusion into the feature extraction process to simplify training steps. At the same time, we will also explore ways to make the model compatible with more other modalities.

## Figures and Tables

**Table 1:** Evaluation results on the RGBT234 dataset. Red, blue, and green represent the first, second, and third place, respectively.

	APFNet	TFNet	DAFNet	DMCNet	MANet++	SiamCDA	ViPT	JMMAC	MaCNet	MACFT
PR (↑)	0.827	0.806	0.796	0.839	0.795	0.760	0.835	0.790	0.790	0.857
SR (↑)	0.579	0.560	0.544	0.593	0.559	0.569	0.617	0.573	0.554	0.622

**Table 2:** Quantitative comparison against state-of-the-art RGB-T trackers on the VTUAV short-term subset. Red, blue, and green represent the first, second, and third place, respectively.

Trackers	PR	SR	FPS
MACFT	80.1%	66.8%	31.7
HMFT [35]	75.8%	62.7%	30.2
ADRNet [4]	62.2%	46.6%	25.0
mfDiMP [36]	67.3%	55.4%	28.0
DAFNet [13]	62.0%	45.8%	21.0
FSRPN [45]	65.3%	54.4%	30.3

**Table 3:** Quantitative comparison against state-of-the-art RGB-T trackers on the VTUAV long-term subset. Red, blue, and green represent the first, second, and third place, respectively.

Trackers	PR	SR	FPS
MACFT	54.1%	46.7%	31.7
HMFT-LT [35]	53.6%	46.1%	8.1
HMFT [35]	41.4%	35.5%	30.2
ADRNet [4]	17.5%	25.3%	25.0
mfDiMP [36]	31.5%	27.2%	28.0
DAFNet [13]	25.3%	18.8%	21.0
FSRPN [45]	36.6%	31.4%	30.3

**Table 4:** Precision rate and success rate (PR/SR) of MACFT and other trackers under different challenge attribute subsets on the RGBT234 dataset. Red, blue, and green represent the first, second, and third place, respectively.

	DMCNet	ViPT	APFNet	TFNet	MaCNet	DAFNet	JMMAC	MACFT
NO	93.7/70.6	94.5/74.2	96.0/70.5	92.1/69.0	94.0/68.4	83.6/60.0	91.6/70.2	97.3/73.0
PO	89.9/63.0	84.4/63.3	85.8/60.4	80.8/56.7	81.1/56.5	80.1/55.9	79.9/59.4	85.7/63.9
HO	71.5/51.2	77.1/57.2	75.0/51.6	68.7/46.6	71.9/48.5	65.6/42.8	67.8/47.6	77.8/56.1
LI	85.3/59.2	79.1/57.2	86.8/58.5	81.5/54.8	81.4/54.0	82.5/55.1	88.4/61.8	82.7/58.3
LR	83.2/58.1	84.6/61.3	83.8/57.3	81.0/53.8	77.9/53.1	77.6/51.9	75.3/51.4	84.2/58.7
TC	87.0/61.9	88.9/66.2	84.4/59.1	80.1/57.2	81.5/59.0	81.8/57.5	72.7/50.4	82.7/60.7
DEF	75.4/54.9	81.3/63.4	79.4/56.8	71.4/50.9	73.5/51.4	65.1/44.2	68.0/51.2	83.4/63.8
FM	81.0/53.6	89.7/64.8	86.6/55.6	76.1/47.4	80.9/49.2	65.6/41.5	68.0/36.6	85.0/59.6
SC	82.3/59.8	82.6/62.9	83.7/58.5	77.6/56.0	78.3/55.7	73.7/50.9	81.0/60.6	86.1/64.1
MB	79.4/60.3	86.8/67.7	80.1/60.2	71.2/53.3	75.8/57.4	62.9/46.1	74.3/56.7	86.9/65.5
CM	81.2/59.4	85.4/65.0	81.0/59.1	76.0/54.1	75.9/54.2	68.1/47.0	74.2/55.0	88.4/65.2
BC	82.1/54.7	83.7/59.1	81.0/54.1	80.2/50.7	79.6/49.0	78.3/45.7	63.8/44.3	85.0/57.9
ALL	83.9/59.3	83.5/61.7	82.7/57.9	80.6/56.0	79.0/55.4	79.6/54.4	79.0/57.3	85.7/62.2

**Table 5:** Evaluation results of MACFT and its variants on the RGBT234 dataset and the LasHeR dataset.

Trackers	LasHeR		RGBT234	
	PR (↓)	SR (↓)	PR (↓)	SR (↓)
MACFT (B-T)	42.8% (−22.5%)	33.2% (−18.2%)	67.4% (−18.3%)	48.8% (13.4%)
MACFT (B-RGB)	57.8% (−7.5%)	46.4% (−5.0%)	80.0% (−5.7%)	58.2% (−4.0%)
MACFT (DM)	62.2% (−3.1%)	48.7% (−2.7%)	83.7% (−2.0%)	58.9% (−3.3%)
MACFT (DM + CAM)	63.8% (−1.5%)	50.3% (−1.1%)	83.7% (−2.0%)	60.7% (−1.5%)
MACFT (DM + MAM)	63.9% (−1.4%)	50.2% (−1.2%)	83.9% (−1.8%)	60.7% (−1.5%)
MACFT (DM + CAM + COM)	64.3% (−1.0%)	50.7% (−0.7%)	85.3% (−0.4%)	61.6% (−0.6%)
MACFT (w/o-FT)	64.3% (−1.0%)	50.8% (−0.6%)	84.0% (−1.7%)	60.9% (−1.3%)
MACFT	65.3%	51.4%	85.7%	62.2%

**Table 6:** The relationship between the number of MAM modules and the tracking performance; the evaluation results in the table are all tested on the LasHeR dataset.

Number of MAM Blocks	PR	SR
4	64.80%	51.00%
5	64.90%	51.20%
6	65.30%	51.40%
7	65.00%	51.20%
8	64.80%	51.10%

**Table 7:** Inference speed, success rate, and corresponding model parameters using MACFT and its variants on LasHeR (* represents the maximum number of trainable parameters in three-stage training, excluding frozen network layers).

Trackers	Speed (FPS)	Params *	SR
MACFT	33.3	59.6 M	65.3%
MACFT (DM + CAM + COM)	35.5	59.6 M	64.3%
MACFT (DM + CAM)	50.5	45.4 M	63.8%

## Data Availability

The raw results of our experiments could be available at: https://drive.google.com/file/d/1Fing9GjH6i5RTvRXuNRZ5fWt03DSE_ZO/view?usp=drive_link (accessed on 4 July 2023).

## Abbreviations

**Symbols**	**Explanations**
*Concat*	Means to connect two tensors in the same dimension.
*W_Q_*, *W_K_*, *W_V_*	Refers to the three weight matrices used by the self-attention mechanism in transformer, namely: query, key, and value.
*d*	Refers to the dim of *W_Q_* and *W_Q_*
*softmax*	Refers to the normalized exponential function; the function is to make the range of each element mapped between (0, 1).
*KL*	Refer to the Kullback–Leibler divergence calculation.
*DR*	Refers to operations that use linear layer dimensionality reduction.
*softArgmax*	Refers to finding the index corresponding to the maximum value through *softmax*.
